# Premature Calcification of Costochondral Cartilage: A Scoping Review of the Literature

**DOI:** 10.7759/cureus.75328

**Published:** 2024-12-08

**Authors:** Jun Jie Benjamin Seng, Zhen-Bing Christine Kho, Navpreet Kaur

**Affiliations:** 1 Medicine, MOH Holdings Private Limited, Singapore, SGP; 2 Family Medicine, SingHealth Polyclinics, Singapore, SGP; 3 Medicine, SingHealth Regional Health System PULSES Centre, Singapore, SGP

**Keywords:** calcification of costochondral cartilage, calcified costochondral cartilage, costochondral cartilage calcification, premature calcified costochondral cartilage, premature costochondral cartilage calcification

## Abstract

Premature costochondral calcification is a rare finding occurring in young patients and is associated with metabolic and endocrinological conditions. Significant heterogeneity exists with regard to its definition and assessment. This scoping review aimed to summarize the prevalence, risk factors, etiology, evaluation, and management of patients with premature costochondral calcification. A scoping review was conducted in PubMed, Embase, CINAHL, CENTRAL, Scopus, and Web of Science, as well as grey literature databases, including OpenGrey and Google Scholar up to 7 December 2024 in accordance with the PRISMA for Scoping Reviews (PRISMA-ScR) checklist. Keywords related to premature calcification of costochondral cartilage were used for the literature search. A narrative review of the prevalence, risk factors, etiology, evaluation, and management of patients with premature costochondral calcification was presented. Of 152 citations, 52 articles were included in this review. Premature costochondral calcification was defined as radiological findings of costochondral cartilage calcification in a patient aged 40 years old or less. The prevalence of premature costochondral cartilage calcification ranges from 0% to 100%, with increasing incidence with age. Common conditions associated with premature costochondral cartilage calcification include hyperthyroidism, trauma/fracture, familial chondrocalcinosis, malignancy, porphyria, and Tietze syndrome. Risk factors associated with premature costochondral calcification include older age, female gender, and history of menstrual disorders. Recommended workup for premature costochondral calcification includes thyroid panel, serum calcium and phosphate, renal profile, liver profile, and vitamin D levels. Premature costochondral calcification remains a radiological finding that warrants careful medical assessment given its association with multiple pathological conditions. More research is required to understand its underlying pathophysiology, optimal initial assessment, and follow-up.

## Introduction and background

The thoracic wall skeleton comprises 12 pairs of ribs, which articulate with the sternum anteriorly via costochondral cartilages [1]. There are a total of 10 costochondral cartilages of which the first seven cartilages articulate with the sternum via sternocostal joints. The remaining three cartilages are connected to form the costal margin via interchondral synovial joints [1]. Collectively, they render stability to the sternoclavicular joint during movement and permit mobility of the chest wall [2].

Calcification of costochondral cartilages occurs via a complex process where secretion of alkaline phosphatase by chondrocytes residing in the cartilage leads to hydrolysis of phosphates to free ions. Subsequently, they combine with soluble calcium within the tissue matrix and precipitate, resulting in calcification [2]. Detection of costochondral calcification on radiographs occurs when there is significant calcification of the costochondral cartilage. It is well established that calcification of costochondral cartilages increases with age [3,4] where its prevalence ranges between 53% and 100% among patients after the sixth decade of life [2,5]. Calcification of the costochondral cartilage is uncommon among patients less than 30 years old. [6] Premature calcification of the costochondral cartilages has been associated with a wide range of pathological conditions, including endocrinological conditions such as hyperthyroidism [7] and malignancies [6]. With increasing accessibility and utilization of chest radiological investigations globally, which may lead to increased pick-up of patients with premature calcification of the costochondral cartilages, a greater understanding of the implications and its management is essential.

Currently, premature calcification of costochondral calcification is a poorly understood phenomenon with varying prevalence. This scoping review aims to summarize the literature on premature calcification of costochondral cartilage in relation to its definition, epidemiology, risk factors, clinical presentation and findings, associated conditions, initial investigation, and management.

## Review

Methods

Protocol and Registration

The search protocol is registered on Open Science Framework, and supplementary files in this review are available at https://osf.io/na3s5/.

Information Sources and Search Strategy

A scoping review of the literature was conducted in PubMed, Embase, CINHAL, CENTRAL, Scopus, and Web of Science, as well as grey literature databases, including OpenGrey and Google Scholar up to 7 December 2024. Due to the expected scarcity of literature related to the topic, a scoping review was performed, instead of a systematic review, to provide a map of the existing literature [8]. Keywords related to premature calcification of costochondral cartilage and calcified costochondral cartilage were used in the search engine of each database. Full details of the search strategy are enclosed in Supplementary File 1.

Eligibility Criteria and Selection Process

As there is significant heterogeneity in definitions of premature calcification of costochondral cartilage related to the age cut-off, we included all articles that evaluated the definition, epidemiology, risk factors, clinical presentation and findings, associated conditions, initial investigation, and/or management of calcified costochondral cartilages. Study designs included randomized controlled trials, observational studies, case series, case reports, and cross-sectional studies. Additionally, data from abstracts from conference proceedings were included due to the expected scarcity of literature on premature costochondral calcification, to enhance the comprehensiveness of the scoping review. We excluded irrelevant systematic reviews, scoping reviews, and non-English. Two reviewers (JJBS and ZBCK) performed the initial screening of articles, and all discrepancies were arbitrated with a third author (NK). A pilot screening exercise was performed for the first 50 articles to ensure congruency of article screening, and the rate of agreement was 94%. Additionally, hand-searching of references of all included articles were performed.

Data Collection Process and Data Items

Data collected included classification methodologies of costochondral calcification, as well as definition, epidemiology, risk factors, clinical presentation and findings, associated conditions, initial investigation, and management of premature calcification of costochondral cartilages. The data extraction process was performed by two independent reviewers (JJBS and ZBCK), and all discrepancies were arbitrated with a third author (NK). With regard to missing data, two email reminders were sent to authors to request the information. If there were no updates after email reminders, the information was labeled as unavailable. Data imputation was not performed for this review.

Effect Measures and Synthesis Methods

A narrative review was performed for the definition, epidemiology, risk factors, clinical presentation and findings, associated conditions, initial investigation, and management related to premature costochondral calcification. The prevalence of costochondral cartilage calcification and premature costochondral cartilage calcification was presented using world map graphics. Additionally, using information synthesized from the review, a workflow for the initial assessment and evaluation of patients with premature costochondral cartilage calcification was proposed for primary care physicians.

To evaluate the suitability of meta-analyses for this review, the clinical and methodological heterogeneity of included studies was examined by two independent reviewers (JJBS and ZBCK). Clinical heterogeneity refers to variations in the characteristics of the patient population, study intervention, and/or outcomes. On the other hand, methodological heterogeneity refers to variation in study design and risk of bias. Due to the expected heterogeneity of included studies, meta-analyses were not conducted.

Results

**Figure 1:**
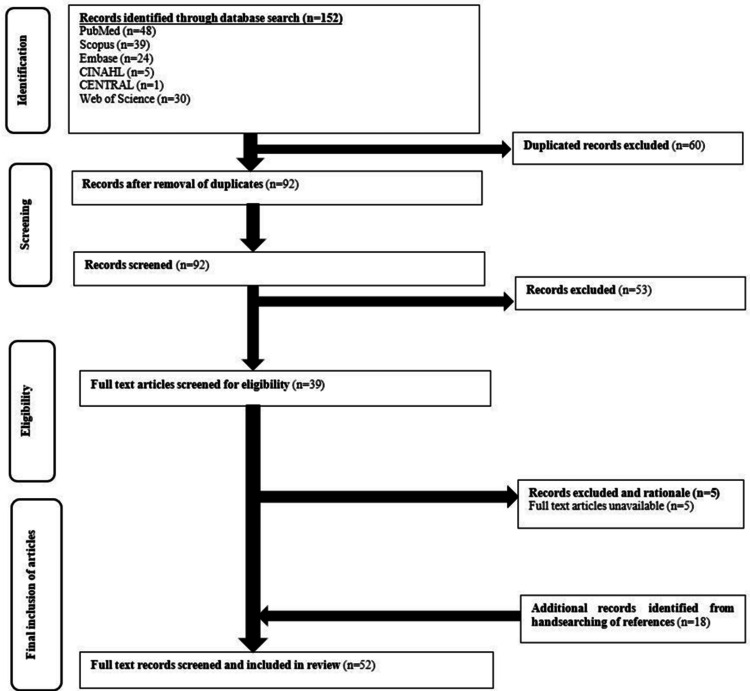
Flowchart for inclusion and exclusion of articles

Figure 1 depicts the flowchart for the inclusion and exclusion of articles. A total of 52 articles were included in the initial search. Details related to included studies were included in Supplementary File 2.

Classification of Costochondral Calcification

Table 1 shows the classification frameworks used for characterizing the severity and nature of costochondral classification. Across the included studies, the most common classification methodology used for assessing the degree of calcification was using a four-stage grading, where stage 0 represents no calcification, while stage 3 represents almost complete/complete calcification (n=3) [9-11]. In contrast, the most common classification methodology used to characterize the nature of costochondral calcification was segregating them into four main categories, namely, “peripheral”, “central”, “mixed”, or indifferent” (n=4) [12-15].

**Table 1:** Definitions related to costochondral calcification by degree and characteristics of calcification

Classification of costochondral calcification		Imaging modality	Number of studies	Reference
Degree of calcification				
1	Stage 0: No calcification.	Chest X-ray, computed tomography scan of chest	3	[9-11]
	Stage 1: Recent onset of calcification <50%.			
	Stage 2: Incomplete but 50% or more calcification.			
	Stage 3: Almost complete or complete calcification.			
2	0 (0%)	Computed tomography of the chest	2	[16,17]
	1 (1-25%			
	2 (26-50%)			
	3 (51-75%)			
	4 (76-100%)			
	-Levels 3 and 4 are considered severe calcification.			
3	Complete calcification: whole cartilage from the coastal to the sternal end was calcified area-wide.	Chest X-ray	2	[18,19]
	Intensive/advanced calcification: at least 50% of the area is involved, independent of calcification, which is gender differences related.			
	Negative: only linear calcification at the costochondral border.			
4	0: no calcification.	Chest X-ray	1	[20]
	+: short linear and ring-like, thin calcifications or small specks of calcium at the distal ends of the lower ribs.			
	++: granular calcifications of the cartilages with or without additional bizarre-shaped calcifications at the distal ends of the ribs.			
	+++: calcifications of the costal cartilages in the shape of densely calcified, curved bands in continuation with the ribs.			
5	Grade 0 - No calcification.	Chest X-ray	1	[1]
	Grade 1 - Traces of calcification.			
	Grade 2 - Calcification along the single margin of the costal cartilage.			
	Grade 3 - Calcification along both the margins of the costal cartilage			
	Grade 4 - Prominent central or bifid calcification.			
	Grade 5 - Mixed – Marginal and central calcification < 50% of calcification.			
	Grade 6 - Mixed 50-74% of calcification.			
	Grade 7 - calcification around 75% and above.			
6	Grade 1: barely visible punctate calcification at end of ribs.	Chest X-ray	1	[7]
	Grade 2: mild calcification coming to a point but not directed upward.			
	Grade 3: moderate calcification curved and directed upward but not interconnecting or reaching the sternum.			
	Grade 4: heavy calcification, interconnecting and extending to the sternum.			
7	Number of ribs of left side with costochondral cartilage calcification	Chest X-ray	1	[21]
	0-1			
	2-Mar			
	4-May			
	6-Jul			
8	Stage 1 (0-25%)	Computed tomography of the chest	1	[15]
	Stage 2 (26-50%)			
	Stage 3 (>50%)			
Nature of calcification				
1	- Peripheral—where calcification presents on inferior	Chest X-ray	4	[12-15]
	or superior margins of cartilage.			
	- Central—where calcification mainly presents on the			
	central part of cartilage.			
	- Mixed—combination of the above two (peripheral and central).			
	- Indifferent—calcification pattern which cannot be distinct into any above categories.			
2	Type I. – peripheral pattern (P), characterized by ossification of the inferior and superior costal cartilage margin.	Chest X-ray, computed tomography scan of the chest	2	[22,23]
	Type II.			
	a) Central lingual pattern (Cl), characterized by pyramidal-shaped central tongues of ossification beginning in the fossae costarum.			
	b) Central globular pattern (Cg), consisting of centrally-placed, smoothly-contoured globules of ossification.			
	c) Central lingual and globular pattern (Clg).			
	Type III – Mixed (peripheral and central) pattern (Mix).			
	Type IV – Indifferent pattern (Ind) – incipient calcification without differentiation into sex-specific pattern.			
3	Central: Clustered towards the core of the cartilage.	Computed tomography of the chest, ultrasound	2	[24,25]
	Marginal: Prominent near the peripherals (marginal).			
	Granular: Disseminated throughout the cartilage.			
4	Grade 1: Mild (flecks of calcification at the costochondral junction).	Chest X-ray	1	[3]
	Grade 2: Moderate (calcification extending into costal cartilage).			
	Grade 3: Marked (widespread calcification in at least 6 costal cartilages).			
5	Type A: Marginal square bracket type.	Chest X-ray	1	[26]
	Type A_1_: Marginal linear type.			
	Type B: Central and tongue-shaped type.			
	Type C: Mixed calcification.			
6	Type I – Marginal.	Chest X-ray	1	[27]
	Type II – Central.			
	Type III – Central type but of parallel linear shadows situated centrally.			
	Mixed type – A combination of type I, II, or III or different ribs in the same individual.			
7	+: Short linear and ring-like, thin calcifications or small specks of calcium at the distal ends of the lower ribs.	Chest X-ray	1	[4]
	++: Granular calcifications of the cartilages with or without additional bizarre-shaped calcifications at the distal ends of the ribs.			
	+++: Calcifications of the costal cartilages in the shape of densely calcified, curved bands in continuation with the ribs.			
8	Type A – Centrally placed, smoothly contoured globules of calcification in the costal cartilages. The cartilage in apposition to the fossae costarum and the sternocostal joints can be heavily calcified.	Chest X-ray	1	[14]
	Type B – Pyramidal central tongues of calcification extending medially from the fossae costarum. A “crab claw” configuration of central calcification may also be seen.			
	Type C – Differs from Types A and B by showing central, fragmental calcification. The foci of calcification are often small, irregular, and sharply angular. In Type C, the pyramidal tongue of calcification is absent.			
	Type D – Mineralization at the fossae costarum and the sternocostal joints. Spurs of calcification may extend from the fossae costarum along the superior border of the cartilages. Types A to D are considered female patterns of calcification.			
	Type E – Characterized by heavy calcification of the superior and inferior margins of the costal cartilages. The sternocostal joints are almost always calcified.			
	Type F – Resembles “Swiss cheese” or a “honeycomb” with sheet-like centrally located calcification and regularly arranged, small, round, radiolucent defects.			
	Type G – Massive costal cartilage calcification with no mineralization at the sternocostal joints.			
	Type H – A finely granular “salt and pepper” appearance of costal cartilage calcification in the lateral thirds of the costal cartilage in a horizontal linear distribution, and no mineralization at the sternocostal joints.			
9	“Zero”: No calcification.	Chest X-ray	1	[5]
	“Thirty”: Minimal calcification.			
	“Sixty”: Medium calcification.			
	“Ninety”: Marked or maximum calcification.			
10	- Without calcification.	Computed tomography of the chest	1	[11]
	- Limited to the second costal notch.			
	- Peripheral linear calcification.			
	- Central coarse granular calcification.			

Definitions of Premature Costochondral Calcification Used in Literature

With regard to definitions of premature costochondral calcification, significant heterogeneity exists in the literature. The most common age limits used are “30 years and below” [18,19,28,29] and “40 years old and below” (Table 2). The justification for the age cut-offs was often derived from individual studies, which evaluated the prevalence of costochondral calcification.

**Table 2:** Definitions of premature costochondral calcification used in the literature

Definition of age cut-offs for premature costochondral calcification	Number of studies	References
40 years old and below	2	[6,30]
35 years old and below	1	[6]
30 years old and below	4	[18,19,28,29]
20 years and below	1	[31]
13 years old and below	1	[32]

In this review, the operational definition of premature costochondral calcification was defined as “radiological calcification of costochondral cartilage in a patient aged 40 years and below”. A broader age cut-off was used to avoid missing diagnoses in patients due to its significant association with pathological conditions. In addition, costochondral cartilage calcification generally had a low prevalence among patients aged 40 years and below.

Epidemiology of Costochondral Calcification and Premature Costochondral Calcification

**Figure 2:**
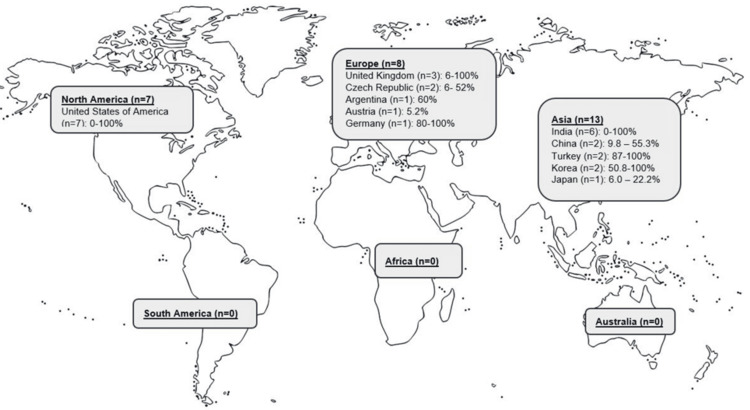
World map depicting the global prevalence of costochondral calcification *Case studies and case series were excluded from inclusion in the world map.

A total of 28 studies (excluding case reports and case series) evaluated the prevalence of costochondral calcification (Figure 2). The majority of the studies were conducted in Asia (n=13), Europe (n=8), and North America (n=7) (Figure 2). There were no studies performed in South America, Africa, or Australia. Of note, the countries with the highest number of studies included the United States of America (n=7) [5,7,27,30,31,33,34], India (n=6) [1,2,10,12,19,26], and the United Kingdom (n=3) [3,14,20]. The overall prevalence of costochondral calcification ranged from 0% and 100%, with similar findings reported in Asia (0-100%), North America (0-100%), and Europe (5.2-100%).

**Figure 3:**
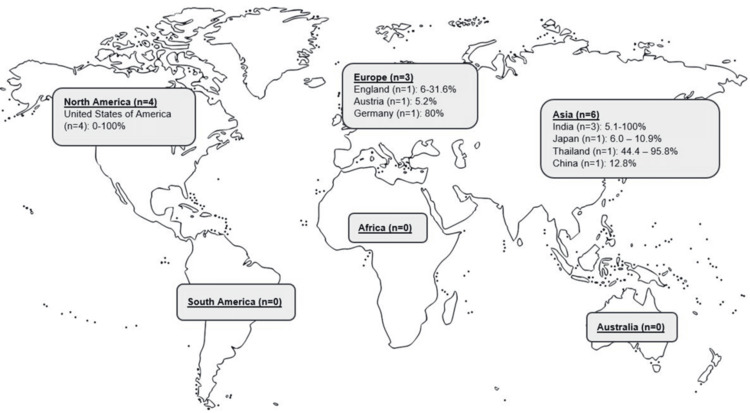
World map depicting the prevalence of premature costochondral calcification *Case studies and case series were excluded from inclusion in the world map.

Specifically for premature costochondral calcification, 13 studies evaluated its prevalence (Figure 3). Most of the studies were conducted in Asia (n=6), North America (n=5), and Europe (n=3). With regards to the countries with the highest number of studies, this included the United States of America (n=4) [5,7,30,31] and India (n=3) [10,12,26]. Similarly, there were no studies conducted in South America, Africa, nor Australia. The prevalence of premature costochondral cartilage calcification ranged from 0% 100%, specifically 0-100% in North America, 5.2-80% in Europe, and 5.1-100% in Asia.

There were four cadaveric studies that evaluated premature costochondral cartilage calcification where its prevalence ranged from 6.0 to 22.5% [13,14,25,35].

Risk Factors Associated With Costochondral Cartilage Calcification

Risk factors associated with costochondral calcification can be broadly divided into factors related to patient demographics, practices, and clinical comorbidities. The most well-studied factors related to patient demographics included older age [1-5,9-13,16,17,20,22,24,35-39] and female gender [4,16,17,20,24,37,38]. Other factors include increased exercise [16], increased fruit intake [16], and a history of osteoarthrosis [20].

Specifically for premature calcification of costochondral cartilages, associated risk factors include older age [26,30,39], females [30], and patients with menstrual disorders [40]. Table 3 presents the risk factors associated with calcification of costochondral cartilage.

**Table 3:** Risk factors associated with calcification of costochondral cartilage

Risk factors	Associated with costochondral calcification	Associated with premature costochondral cartilage calcification
Patient demographics	Older age [1-5,9-13,16,17,20,22,24,35-39]	Older age [26,30,39]
	Female gender [4,16,17,20,24,37,38]	Female gender [30]
	Male gender [3,5,41]	
	Increased body mass[16,37]	
	Blacks (vs Whites) [5,37]	
Patient practices	Increased fruit intake[16]	Nil
	Later bedtime [16]	
	Increased exercise [16]	
Clinical comorbidities or variables	History of osteoarthrosis[20]	Menstrual disorders [40]
	Higher fasting blood glucose [37]	
	Higher Hba1c [37]	
	Diabetes mellitus [38]	

Conditions Associated with Premature Costochondral Calcification

The main diseases associated with premature costochondral calcification can be broadly divided into endocrinological, musculoskeletal/rheumatological, malignancies, infection, medication-related, renal causes, medication-related, congenital/rare diseases, and idiopathic causes.

Among the cases reported, hyperthyroidism [6,7], fracture-/trauma-related [42], familial chondrocalcinosis [34,43], porphyria [18,19], and Tietze syndrome [6,44-46] formed the most common conditions associated with premature costochondral cartilage calcification.

Table 4 provides a summarized overview of its associated conditions, the patient’s initial presenting complaint, physical examination, and common blood investigation findings. Detailed information related to patients’ presenting complaints reported in individual studies is reported in Supplementary File 3.

**Table 4:** Premature costochondral calcification: associated conditions, pathophysiology, presenting complaints, physical examination findings, and blood investigations

Disease classes	Associated conditions	Postulated pathophysiology	Total number of cases	Common clinical presentation	Common physical examination findings	Common blood investigation findings	References
Endocrine	Hyperthyroidism	May be related to enhanced production of growth hormone by increased thyroid hormone level	33	Weight loss	Fever	Increased free thyroxine	[6,7]
		Alteration of skeletal metabolism by excessive thyroid hormone		Tremors	Tachycardia	Reduced thyroid-stimulating hormone	
				Neck swelling	Exophthalmos	Positive antibodies to thyroid receptor antagonist	
				Palpitations	Neck swelling		
				Diarrhea	Tremors		
				Increased anxiety/irritability			
				Heat intolerance			
				Altered menstrual cycles			
	Adrenogenital syndrome	Accelerated bone maturation	1	Failure to thrive	Hypotension	Hyponatremia	[47]
				Recurrent vomiting, diarrhea	Hypertension	Hyperkalaemia/hypokalaemia	
				Early virilization	Ambiguous genitalia	High serum 17-hydroxyprogesterone	
				Ambiguous genitalia	Early development of pubic hair, phallic enlargement	Low aldosterone	
					Hyperpigmentation of genitalia and areolae	Elevated renin	
						Low cortisol	
Musculoskeletal/Rheumatological	Cartilage fracture, trauma	Injury to costochondral cartilage	39	Preceding trauma	Tenderness over site of injury		[42]
	Familial chondrocalcinosis	Increased serum calcium/phosphate ratio and localized changes in tissue collagen types, glycosaminoglycans, and lipids may increase dystrophic apatite deposition	32	Acute, recurring attacks of pain, swelling, warmth, and redness in one or more joints	Tenderness, stiffness, swelling, and loss of function in the affected joints	Presence of calcium pyrophosphate crystals in intraarticular tissue	[34,43]
						Positive ANKH gene testing	
	Tietze syndrome	Unclear etiology	7	Pain and swelling around costosternal syndrome	Tenderness and swelling in upper ribs	Generally normal blood investigations	[6,44-46]
		May be related to trauma and infection					
		Involvement of increased vascularity, degeneration with cleft formation and calcifications					
	Chondrodysplasia punctata	Unclear	1	Tenderness or stiffness of joints	Short stature	Genetic testing	[32]
				Recurrent respiratory tract infections	Contractures		
					Prominent forehead, wide-set eyes		
					Rhizomelia		
					Cataracts		
					Underdevelopment of the middle face		
	Diastrophic dwarfism or dysplasia	Unclear	1	Short stature	Shortening of limbs	Genetic testing	[32]
				Delayed motor skills	Scoliosis		
					Hip dysplasia		
					Joint deformities		
					Hernia		
					Cauliflower ear		
					Cleft palate		
					Hitchhiker’s thumb		
					Brachydactyly		
Malignancy	Primary malignancy (e.g. chondrosarcoma)	Arise in costal cartilage	1	Symptoms related to primary malignancy	Clinical findings relevant to primary malignancy	Relevant investigations specific to each malignancy	[6]
		Approximately 10% in the thoracic cage					
		Slow growth					
	Metastatic cancer	Lung cancer: direct invasion	2	Symptoms related to malignancy	Clinical findings relevant to primary malignancy	Relevant investigations specific to each malignancy	[6,44]
		Breast cancer: invasion of costal margin by primary tumor or involved mammary nodes					
		Tumors may secrete proteins with parathyroid hormone-like activity that alter calcium metabolism and increase bone resorption					
Infection	Infections such as disseminated infection, post-operative infection	Extension of pulmonary infection or hematogenous dissemination as a postoperative infection	3	Fever, pain over site/joint	Pyrexia	C-reactive protein, procalcitonin	[6]
				Recent operation	Tenderness over site of investigation	Leucocytosis	
Renal	Chronic renal failure	Altered calcium balance	1	Asymptomatic	Symptoms of fluid overload	Elevated creatinine	[6]
				Reduced urinary output		Other relevant investigations secondary to the etiology of chronic renal failure	
				Haematuria or frothy urine			
Medication-related	Mitral valve replacement and warfarin sodium therapy	Unclear etiology	3	Asymptomatic	Not applicable	No specific blood investigations	[31]
				Recent mitral valve replacement and warfarin			
Congenital/rare conditions	Porphyria	Unclear etiology	15	Non-specific abdominal pain	Altered mental status	Elevated urine porphobilinogen	[18,19]
				Nausea, vomiting	Abdominal tenderness	Genetic testing (if relevant)	
				Myalgia, weakness			
				Haematuria			
				Rashes			
	Keutel syndrome	Unclear etiology	1	Hearing loss	Calcification of ears, nose, larynx, trachea, or ribs	Gene testing	[32,48]
				Recurrent respiratory tract infection	Brachytelephalangism		
					Hypoplastic facial features		
					Intellectual disability		
Idiopathic	Idiopathic	Unclear etiology	2	Usually asymptomatic	Normal examination	Normal blood investigations	[6,28,29]

Details related to the management of individual conditions were not specified as this is beyond the scope of the review, and there exist more detailed references available in the literature to address this matter.

Proposed Workflow for Initial Assessment and Evaluation of a Patient with Premature Costochondral Calcification

**Figure 4:**
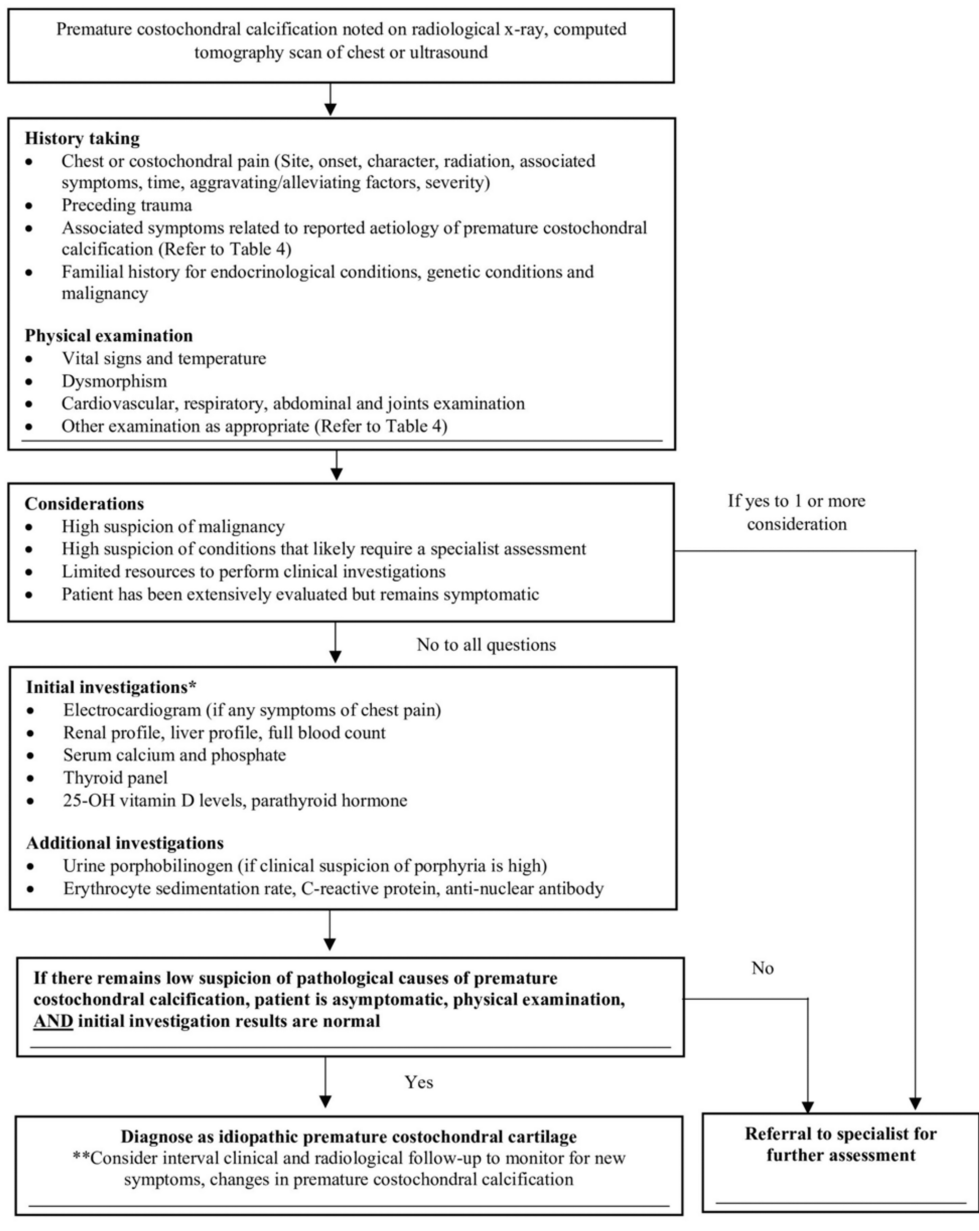
Proposed workflow for patients in primary care with premature costochondral calcification * The recommended list of investigations is non-exhaustive and should be tailored according to the patient.

Figure 4 depicts the proposed workflow for assessing and evaluating a patient with premature costochondral calcification in a primary care setting. A detailed history and physical examination are essential due to the myriad of conditions associated with premature costochondral calcification. For patients who require more urgent specialist care, recommendation for early specialist referral is suggested. For patients who are eligible for initial assessment in primary care, the investigations listed are ubiquitous and aim to target common treatable pathologies such as hyperthyroidism [6,7] and renal failure [6].

For patients with low suspicion for pathological causes of premature costochondral calcifications and are asymptomatic with normal physical examination and initial investigations, consideration for diagnosis of idiopathic premature costochondral cartilage calcification can be made with interval follow-up to monitor for development of new symptoms.

Discussion

This review has summarized the definition, epidemiology, risk factors, clinical presentation and findings, associated conditions, initial investigation, and management of premature costochondral calcification. There were several key research gaps identified from this review. Firstly, studies that evaluated the prevalence of premature costochondral cartilage calcification often do not report its prevalence segregated by age/gender subgroups, as well as details related to pre-existing comorbidities or investigations performed for patients. This renders significant difficulties in assessing age- or gender-specific prevalence and potential underlying causes for premature costochondral cartilage calcification. Additionally, due to the lack of segregation of gender-specific data, it is difficult to evaluate if a gender-specific definition of premature costochondral calcification is required. This is significant given the generally higher rates of costochondral cartilage calcification noted among females in this review and should be considered in future updated reviews [30].

Secondly, there are currently no gold standard classification tools for assessing the degree and pattern of costochondral cartilage calcification. From our review, the main classification methodology utilized was a four-stage grading system for increasing the degree of calcification and four main patterns of costochondral calcification (“peripheral”, “central”, “mixed”, or indifferent”). Consideration should be made for future research involving an expert panel to assess which is the most optimal classification methodology, to standardize reporting in future research in this field.

Thirdly, the optimal preliminary assessment and evaluation of patients were generally poorly reported across studies (Supplementary File 3). While we proposed a workflow that broadly covers common pathological conditions associated with premature costochondral calcification within reasonable limits with caveats for specialist referrals, it is unclear if a conservative approach such as interval radiological surveillance is a reasonable initial management option. Similarly, it remains unclear regarding the follow-up of patients with idiopathic premature costochondral calcification.

In addition, there is currently a paucity of data related to premature costochondral calcification among patient populations in South America, Africa, and Australia. More studies are required to evaluate if there are inter-continent/country differences in the prevalence of premature costochondral calcification.

Limitations

This review is not without limitations. Firstly, the search is limited to English-language articles, and future reviews should consider the inclusion of non-English articles in the review. In addition, despite a relatively comprehensive search strategy, a search of relevant references of included literature and grey literature, potentially relevant articles may still be missed. Thirdly, a significant proportion of studies were derived from patients with very specific presenting complaints such as abdominal pain who underwent abdominal X-ray imaging or cadaveric studies. This in turn may limit generalizability to the general population. In addition, risk of bias analyses were not performed in this review to allow for comprehensive mapping of the literature. Nonetheless, we believe that the results from this scoping review will guide future researchers to perform more targeted future systematic reviews to assess and evaluate premature calcification of costochondral cartilages. More studies are required to evaluate if there have been any temporal trends in the prevalence, associated conditions, and risk factors associated with premature costochondral calcification.

## Conclusions

Premature costochondral calcification remains a radiological conundrum that warrants careful medical assessment given its association with multiple pathological conditions. This review has summarized the prevalence, risk factors, associated conditions associated with premature costochondral calcification. More research is required to understand its underlying pathophysiology, optimal initial assessment, and follow-up.
